# Feasibility and reliability of a smartwatch to detect atrial fibrillation after cardiac surgery: a prospective study

**DOI:** 10.3389/fdgth.2025.1718350

**Published:** 2026-01-16

**Authors:** Konrad Schreier, Michael Borger, Alireza Sepehri Shamloo, Lukas Hofmann, Thomas Schröter, Sandra Eifert, Angeliki Darma, Christian Etz, Sergey Leontyev, Martin Misfeld, Andreas Bollmann, Arash Arya

**Affiliations:** 1 Department of Cardiac Electrophysiology, Heart Center Leipzig, Leipzig, Germany; 2 Department of Cardiac Surgery, Heart Center Leipzig, Leipzig, Germany; 3 Department of Cardiothoracic Surgery, Royal Prince Alfred Hospital, Australia Institute of Academic Surgery, Sydney, NSW, Australia; 4 The Baird Institute of Applied Heart and Lung Surgical Research, Australia Sydney Medical School, University of Sydney, Sydney, NSW, Australia; 5 University Clinic and Polyclinic for Cardiology, University Hospital Halle (Saale), Martin-Luther University Halle-Wittenberg, Halle, Germany

**Keywords:** atrial fibrillation, cardiac surgery, monitoring, screening, smartwatch

## Abstract

**Background:**

Atrial fibrillation, the world's predominant cardiac arrhythmia, frequently emerges as a complication post-cardiac surgery, leading to serious outcomes like strokes, heart failures, and even death. Due to its often-silent nature, detecting it can be challenging. Smartwatches present a potential solution, offering screening that is more rigorous.

**Objective:**

This prospective observational study sought to assess the Withings Scanwatch's efficacy in identifying postoperative atrial fibrillation.

**Methods:**

After cardiac surgery, patients received a Withings Scanwatch. Over a span of 24 h, both the smartwatch's photoplethysmography sensor and standard telemetry kept track of any atrial fibrillation incidents.

**Results:**

At the end of the study, data from 260 patients was available for assessment. Atrial fibrillation was identified in 32 of these patients, either via telemetry or the smartwatch. Our data revealed a sensitivity of 69.0%, specificity of 98.7%, a positive predictive value of 87.0%, and a negative predictive value of 96.2%.

**Conclusions:**

This clinical study is the first to evaluate the photoplethysmography sensor of the Withings Scanwatch, and it shows that the Scanwatch has high a specificity and moderate sensitivity in detecting postoperative atrial fibrillation. Thus, Scanwatch may support the conventional screening for atrial fibrillation, and potentially reducing complications and costs of atrial fibrillation. Because of lower than expected sensitivity this technology cannot replace conventional monitoring in postoperative patients.

## 1 Introduction

Atrial fibrillation (AF) is the most prevalent cardiac arrhythmia globally, particularly among the elderly (1). Furthermore, AF increases the risk of stroke, thromboembolic events, heart failure, and mortality (2). A challenge with AF is that it often presents with no symptoms or very vague ones, leading to late detection (2).

The pathophysiology of atrial fibrillation involves specific structural and electrophysiological mechanisms. The genesis of AF requires a vulnerable atrial substrate as well as a trigger, which usually originated from pulmonary veins (3). The vulnerable substrate is characterised by structural, architectural and electrophysiological abnormalities and can have a large spectrum of origins. The probably most common trigger for AF is ectopic activity localised in the pulmonary veins, which have unique electrical properties and complex fibre architecture (4). Another important trigger for AF is inflammation (5) and that is the reason why AF is not uncommon after cardiac surgery.

Therefore, AF is a clinically relevant condition not only in internal medicine but also in cardiac surgery. Postoperative atrial fibrillation (POAF) is the most common complication following surgical procedures, with a frequency of 20–30% (6). Previously, POAF was believed to be a transitory, self-resolving condition. However, modern understanding associates it with complications such as ischaemic stroke, heart failure, prolonged hospitalisation and death (7, 8). Similar to common AF, POAF often goes unnoticed and tends to recur (9, 10). Therefore, there are many different considerations and approaches on how to avoid these complications. For example, the ESC Guidelines recommend perioperative amiodarone therapy or concomitant posterior pericardiotomy (11). Another approach is an amputation of the left atrial appendage during surgery to prevent thrombus development even before POAF occurs (12). Nevertheless, prompt and efficient detection is crucial to manage POAF effectively and prevent potential serious complications. However, intensive monitoring for example with telemetry is still expensive and time-consuming, and continuous monitoring over a longer time period of more than one week is hardly practical.

As science advances, so does technology, particularly in the area of e-health and wearable technologies such as fitness trackers and smartwatches. These wearables are becoming increasingly popular and consumers have a growing variety of options available to them. One of the most important methods of measuring used by smartwatches is photoplethysmography (PPG) for pulse rate measurement. A measuring technique that has been known for many decades and that is still being researched. One of the latest research subjects is the detection of AF by wearables using PPG.

The first versions of these devices have already been extensively evaluated, but newer and subsequent models still need to be further analysed. One such device that has not been thoroughly examined is the Scanwatch by Withings. The Scanwatch has not undergone formal clinical validation. While some studies have assessed its ability to detect AF (14, 15), to our knowledge, none have explored its use of a PPG sensor for this purpose. Therefore, we undertook a study to assess the reliability and feasibility of the Scanwatch in detecting POAF.

It therefore differs from other studies, such as the AppleHeartStudy (13), which were conducted for AF screening in the general population, particularly with regard to movement and arrhythmia burden. Patients who have undergone heart surgery are less likely to move around in the first few days after the operation and have a higher risk of arrhythmias, which which likely results in a higher arrhythmia burden in this population.

## 2 Methods

### 2.1 Study design and objective

We conducted a comprehensive prospective observational study at the Leipzig Heart Centre, aiming to evaluate the effectiveness and reliability of a commercially available smartwatch in detecting AF in patients who had recently undergone cardiac surgery. For comparison we chose telemetric monitoring as the gold standard for detecting arrhythmias after cardiac surgery. The study was meticulously designed to include a diverse cohort of participants, each of whom provided informed consent prior to their involvement. This research was carefully reviewed and received formal approval from the Ethics Committee of the University of Leipzig, ensuring that all ethical considerations were addressed. Patients were enrolled between November 2021 and January 2023, and inclusion occurred on the day of transfer to the general ward or the following day.

### 2.2 Participants

Eligible participants for this study were individuals aged 18 years and older who had recently undergone cardiac surgery. To be considered for inclusion, participants needed to have an electrocardiogram (ECG) indicating sinus rhythm prior to their surgery, ensuring that their heart rhythm was normal at the baseline. Additionally, they were required to be continuously monitored by telemetry throughout the study period to accurately capture any changes in heart rhythm that might occur postoperatively. We only included the patients after their stay in the intensive care unit, because we wanted to test the watch under real conditions on a normal unit and the gold standard of an ECG can be used for screening in an intensive care unit without any problems, especially because the patients rarely move. We implemented strict exclusion criteria to maintain the integrity of the study. Patients with a documented history of AF were excluded, because in the 2024 guidelines, POAF is described as new onset atrial fibrillation in the immediate postoperative period (11). We also excluded patients, who had implanted cardiac devices such as pacemakers or defibrillators, which could interfere with the detection of AF by the smartwatch. Furthermore, individuals with cognitive impairments were also excluded to ensure that participants could fully understand and comply with the study procedures. This careful selection process was designed to create a well-defined participant pool, enabling us to accurately assess the smartwatch's ability to detect new-onset AF in a postoperative setting.

### 2.3 AF detection methodology: smartwatch vs. telemetry

Smartwatch: After being informed and providing consent, patients were equipped with a Scanwatch provided by Withings. Patients were permitted to wear the watch on either arm according to individual preference. They were advised to ensure continuous skin contact for accurate readings. Patients were allowed to remove the watch when temporarily leaving the ward, as telemetry was not active during these periods. They were instructed to wear the watch for 24 h without interruption if possible. We decided on this time period because some patients were only monitored by telemetry for 24 h after being discharged from the Intensive Care Unit (ICU) or Intermediate Care (IMC). A second reason for this 24-hour period was the limited number of watches available and this approach was chosen to maximize patient enrolment within the study period. The Scanwatch recorded the heart rate every 10 min using a PPG sensor and scanned the data for AF indicators.

The method of PPG works by emitting light of a specific wavelength, usually in the visible or near-infrared spectrum, into the tissues, typically the skin. The Scanwatch's PPG system utilizes green light. As the light penetrates the tissue, it interacts with various components, primarily blood. A photosensor, positioned either adjacent to or in the same device as the light source, detects the light that is either reflected off or transmitted through the tissue. The information captured by the photosensor is processed to generate a photoplethysmogram, a signal that displays a characteristic waveform (16). This waveform fluctuates in response to the changes in blood volume within the tissue during each cardiac cycle. The resulting pattern can be analysed to draw conclusions about the peripheral pulse, which provides insights into cardiovascular health. PPG is particularly valuable due to its simplicity, low cost, and ability to provide real-time monitoring. This technique is now widely integrated into wearable devices, such as smartwatches and fitness trackers, to continuously monitor heart rate, blood oxygen saturation, and other vital signs. It can also be used to detect irregularities in heart rhythms, such as AF, by analysing the variability and consistency of the pulse wave over time. This data was then synced and stored using Withings’ Health Mate App. By continuously monitoring the heart's rhythm, the device can alert users to potential irregularities, prompting further medical evaluation if necessary. The smartwatch's PPG-recordings and analyses were not visible on the smartwatch itself or in the Health Mate App. The app only gave information about the patients’ heart rate while wearing the smartwatch. As soon as the app registered 10 suspicious measurements within 24 h, a notification appeared on the connected smartphone. The design of the Scanwatch's PPG system reflects a balance between technological sophistication and user convenience. Compared to most other smartwatches, the Scanwatch does not have a touchscreen but instead an analogue display with a small screen and is operated entirely by a crown. The Scanwatch has received CE certification in Europe and FDA approval in the United States. On the Website of Withings they state that personal data is processed in accordance with applicable privacy and personal data protection laws, as set out in the new European General Data Protection Regulation (GDPR).

Telemetry: Telemetry monitoring utilized a 5-lead ECG. Five electrodes were appropriately positioned on the patient's torso. The telemetry system captured data from various leads, including leads I-III, aVL, aVR, aVF, and a chest wall lead V. Notably, the electrode for the chest wall lead V was slightly repositioned to the left due to the dressing of the sternotomy wound. Typically, leads II and V were monitored with heart rate alarm thresholds set to <50/min and >135/min. However, medical staff had the discretion to adjust these limits. In comparison to the smartwatch, monitoring via telemetry was performed continuously. Any occurrences of tachyarrhythmia or AF that persisted ≥30 s were documented by the attending physicians or nurses, and we sourced our data from these notes. This means that the AF episodes did not need to be confirmed by a 12-lead ECG. The physicians themselves decided whether they wanted or not to confirm the diagnosis of AF with a 12-lead ECG or long-term ECG.

### 2.4 Statistical analysis

Continuous data were presented in this study as mean values accompanied by their corresponding standard deviations (SD). For categorical or binary data, results were expressed as absolute numbers along with their relative frequencies, reported in percentages. For continuous variables, group differences were analysed using the *t*-test after verifying the normal distribution of the data. In cases of categorical data, the Chi-Square Test was employed to evaluate the association between groups by comparing the observed frequencies to those expected under the null hypothesis. Both tests were two-sided to account for the possibility of differences in either direction and a *p*-value of less than 0.05 was considered the threshold for statistical significance. For time-to-event analyses, such as examining the occurrence of AF over the study period, nonparametric estimates were calculated using the Kaplan–Meier method. The log-rank (Mantel-Cox) test was utilized to compare the survival curves between groups, determining whether there was a statistically significant difference in the event times. All statistical analyses were conducted using SPSS software, specifically Version 27.

## 3 Results

**Figure 1:**
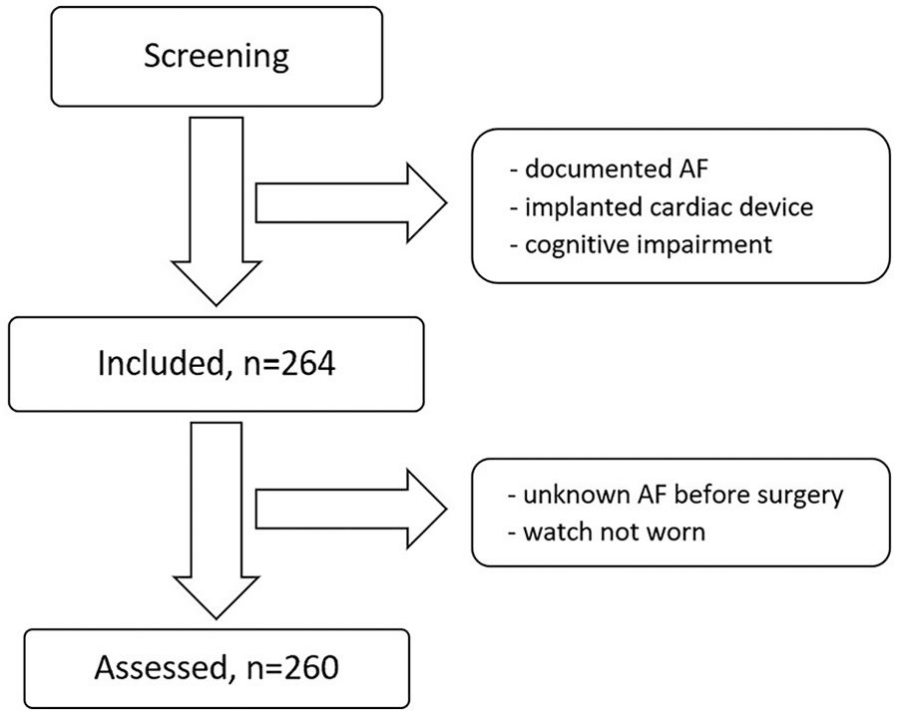
Inclusion process.

Between November 2021 and January 2023, we enrolled 264 patients for our study. However, 4 participants were later excluded, leaving us with 260 datasets that underwent statistical analysis. The reasons for exclusion were the patient's decision not to wear the watch or a known but undocumented AF before the surgery. A short overview about the process of screening, inclusion and assessment is given in Figure 1. Out of these 260 patients, telemetry identified 29 with POAF while they wore the Scanwatch. From this subset, the Scanwatch detected “signs of AF” in 20 patients. Interestingly, 3 patients displayed “signs of AF” on the Scanwatch, though telemetry did not confirm this diagnosis. Consequently, the Scanwatch demonstrated a sensitivity of 69% and a specificity of 98.7%. Given the AF prevalence of 9% in our study population, we determined a positive predictive value (PPV) of 87.0% and a negative predictive value (*N*PV) of 96.2%. All these main results are also summarized in Table 1.

**Table 1:** Accuracy of the smartwatch screening.

Screening methods		Smartwatch screening		Total (% of 260)
		No AF (% of 260)	AF (% of 260)	
Telemetry screening	No AF (% of 260)	228 (87,7%)	3 (1,1%)	231 (88,8%)
	AF (% of 260)	9 (3,5%)	20 (7,7%)	29 (11,2%)
Total (% of 260)		237 (91,2%)	23 (8,8%)	260 (100%)

Table 2 displays the baseline patients’ characteristics. Information for this table was driven from admission records, surgical reports, and discharge documentation. It reveals a significant age difference between patients diagnosed with AF and those without. Moreover, AF patients typically had higher CHA2DS2-VA-Scores. To predict postoperative atrial fibrillation, a binary logistic regression model was constructed with AF as the dependent variable and the CHA2DS2-VA score as a predictor. The CHADS-VA score was significantly associated with the development of postoperative atrial fibrillation (*β* = 0.29, OR 1.33; 95% CI 1.03–1.72; *p* = 0.031). This corresponds to an approximate 33% increase in the odds of postoperative atrial fibrillation for each additional point in the CHA2DS2-VA score. The predicted probability of postoperative atrial fibrillation increased steadily with higher CHA2DS2-VA scores, ranging from approximately 4% for a score of 0 to about 31% for a score of 8 (Table 3). Last but not least, were more likely to have more than 1 surgical indication, what means that the patients received at least 2 of the three surgical procedures: CABG, valve surgery or aortic replacement.

**Table 2:** Baseline patient characteristics*.*

Variable	With AF	Without AF	Total	*p*-Value
Age (in years)	69,76 (±9,716)	64,92 (±10,094)	65,46 (±10,15)	0,016
CHA2DS2-VA-Score	3,83 (±1,58)	3,16 (±1,54)	3,23 (±1,56)	<0,05
Female	6 (20,7%)	48 (20,8%)	54 (20,8%)	0,991
CABG	11 (37,9%)	104 (45%)	115 (44,2%)	0,466
Valve surgery	8 (27,6%)	84 (36,4%)	92 (35,4%)	0,343
> 1 surgical indication	10 (34,5%)	38 (16,5%)	48 (18,5%)	0,028
Diabetes mellitus I/II	10 (34,5%)	69 (29,9%)	79 (30,4%)	0,614
Obesity	14 (48,3%)	81 (35,1%)	95 (36,5%)	0,170
Coronary artery disease	24 (82,8%)	165 (71,4%)	189 (72,7%)	0,178
Arterial hypertension	25 (86,2%)	191 (82,7%)	216 (83,1%)	0,625
Hyperlipidemia/-cholesterinemia	17 (58,6%)	162 (70,1%)	179 (86,8%)	0,217
Chronic renal failure	6 (20,7%)	27 (11,7%)	33 (12,7%)	0,198
COPD	1 (3,4%)	7 (3%)	8 (3,1%)	0,904
OSAS	0 (0%)	12 (5,2%)	12 (4,6%)	0,088

AF, atrial fibrillation; CABG, coronary artery bypass graft; COPD, chronic obstructive pulmonary disease; OSAS, obstructive sleep apnea syndrome. Data as mean value (±SD) or as absolute number (percentage within the group).

**Table 3:** Risk of AF depending on CHA2DS2-VA-Score.

CHA2DS2-VA-Score	AF risk
0	4,4%
1	5,8%
2	7,6%
3	9,8%
4	12,7%
5	16,2%
6	20,4%
7	25,5%
8	31,3%

A total of 145 bypass operations were performed, in which a total of 327 bypasses were placed. Heart valve operations were performed in a further 139 operations, 24 of which involved reconstruction. A total of 114 aortic valves, 27 mitral valves and 2 tricuspid valves were operated on. 4 patients underwent surgery on 2 valves. Five patients underwent aortic replacement. A total of 74 patients did not undergo cardioplegia and were treated with beating heart surgery. In addition, 46 patients did not have sternotomy as the access route but underwent mini-thoracotomy. To evaluate whether AF was influenced by surgical access type and whether an interaction between access type and the CHA2DS2-VA-Score existed, a logistic regression model was constructed that included CHA2DS2-VA-Score, access type (sternotomy vs. minimally invasive), and their interaction term. In the multivariable model, neither the surgical access (OR 0.64; 95% CI 0.08–5.27; *p* = 0.68) nor the interaction between CHA2DS2-VA-Score and access type (OR 1.15; 95% CI 0.66–2.00; *p* = 0.62) showed a significant association with the occurrence of AF. When comparing AF incidence directly between groups, AF occurred in 11.8% of patients undergoing sternotomy and in 9.8% of those undergoing minimally invasive approaches. This difference was not statistically significant (z = −0.49, *p* = 0.63). Overall, these findings indicate that, in this cohort, sternotomy was not associated with an increased risk of postoperative atrial fibrillation compared with minimally invasive surgical access, and no significant interaction between surgical access and the CHA2DS2-VA-Score was observed.

On average, patients spent about 1.2 days in either the ICU or IMC post-operatively and were equipped with the Scanwatch after 1–4 days after surgery (Mean value: 1,45 days). The app also showed how many episodes of AF were detected by the Scanwatch. On average, around 2.1 AF episodes were recognised for each patient that the Scanwatch assessed as positive. After atrial fibrillation occurred, most patients initially received conservative therapy in the form of electrolytes or beta-blockers (*n* = 27). If this did not result in spontaneous conversion, electrical cardioversion was usually applied next (*n* = 14), and if this was unsuccessful, amiodarone treatment was initiated (*n* = 8). However, three patients did not receive any specific treatment for atrial fibrillation.

**Figure 2:**
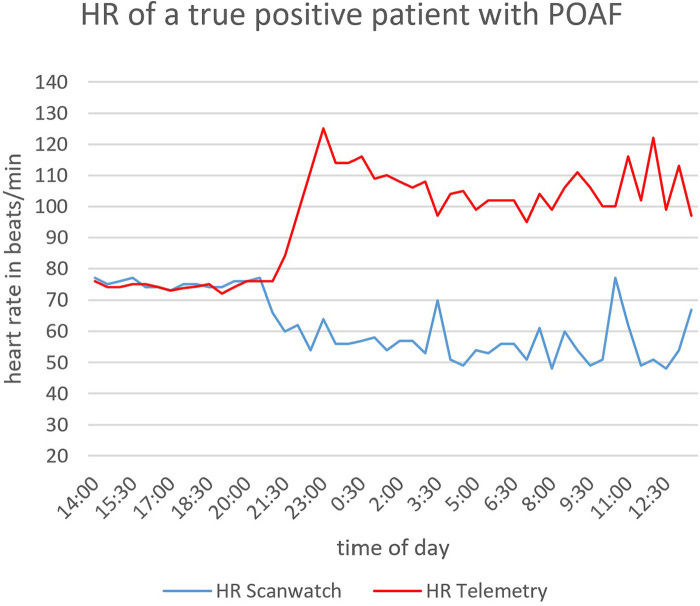
HR measurement over 24 h. HR, heart rate.

**Figure 3:**
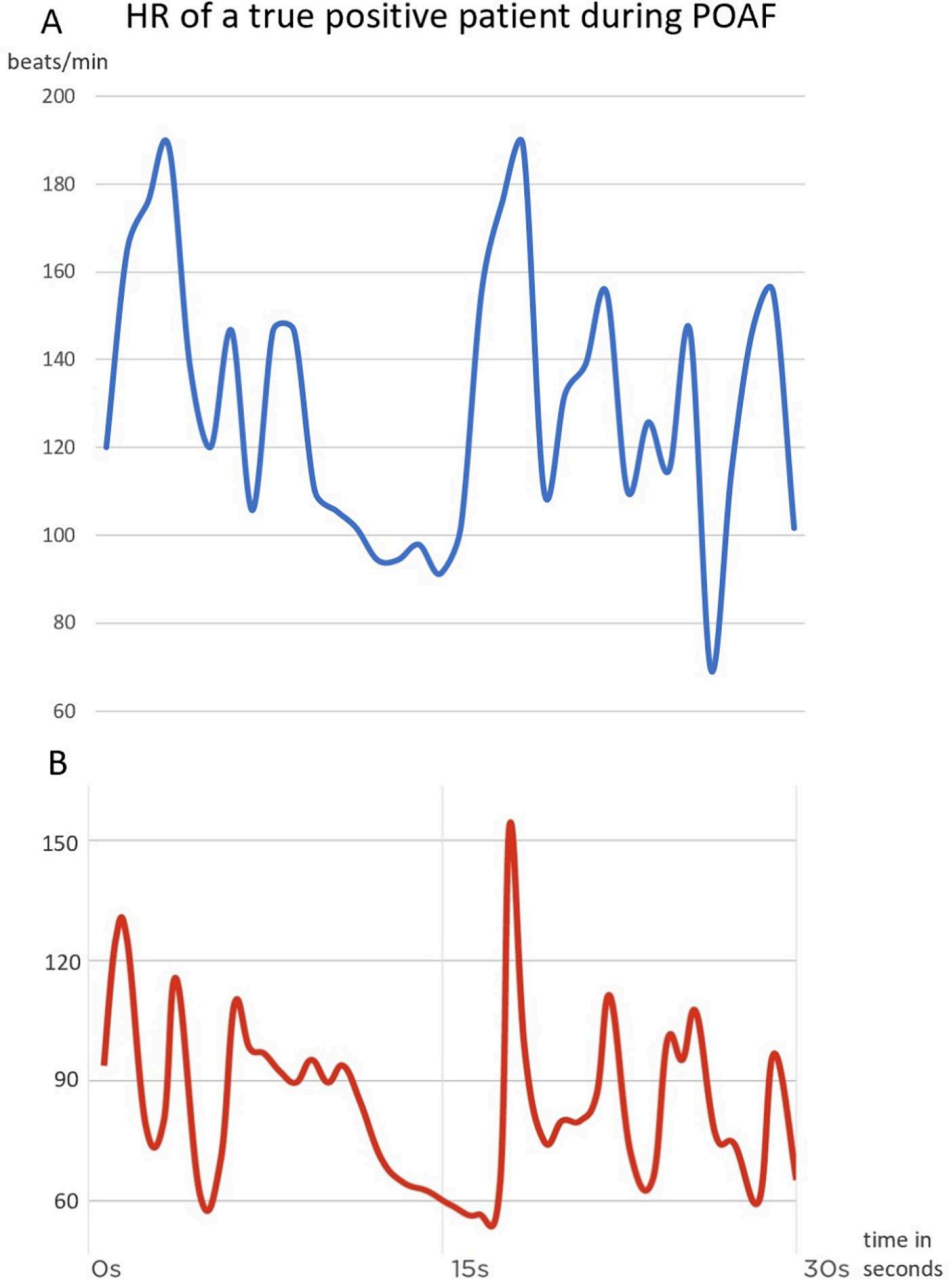
HR measurement during AF; HR, heart rate; BPM, beats per minute; part A of the graph shows the HR change measured by the telemetry and part B the measurements of the scanwatch during a time period of 30 s.

We also compared the heart rate of a true positive patient as an example. Figure 2 shows the heart rate values measured by telemetry and the Scanwatch during 24 h of monitoring of one and the same patient. Figure 3 shows the measured heart rate of the same true positive patient during 30 s of AF. Throughout their hospital stay, 63 patients exhibited POAF. The average post-surgical hospitalization duration was 9.3 days. Notably, a significant difference in the duration of hospital stay was observed between the two patient groups, as depicted in Figure 3. Patients who did not develop POAF tended to have considerably shorter hospital stays. By the 10-day mark, nearly 90% of these patients had been discharged, reflecting a quicker recovery and return to normal health. In contrast, fewer than 70% of patients who experienced POAF were discharged by the same time point, indicating that those with POAF generally required longer hospitalization. This extended stay may be attributable to the need for additional monitoring, treatment, and management of complications associated with POAF, further highlighting the clinical impact of this condition on postoperative outcomes.

## 4 Discussion

Our study demonstrates that using a smartwatch to detect POAF is both practical and reliable. To the best of our knowledge, this is the first study to specifically investigate the detection of AF using the PPG sensor in the Withings Scanwatch. Prior to our research, the only available data on AF detection with this device came directly from studies conducted by Withings itself. Withings reported a sensitivity of 98% and a specificity of 93% in their investigation. However, we were unable to locate specific details regarding the performance metrics or the underlying study data. Additionally, exact information on the algorithm used to evaluate the PPG signal has not yet been made publicly available. While the sensitivity and specificity figures reported by Withings are higher than those observed in our study, the two investigations are not entirely comparable. One key difference is that our study focused on a specific patient group, whereas in the Withings study, about 20% of the recordings were considered inconclusive and were subsequently excluded. Another factor that may account for the difference in outcomes is that our study was designed to reflect real-life clinical scenarios rather than being conducted under tightly controlled conditions. For example, we provided participants with detailed instructions on how to use the smartwatch but did not supervise or influence how they wore it or for how long. This approach likely makes our results more representative of real-world usage and outcomes. The underlying reasons for the Scanwatch's detection of AF episodes in these 3 cases remain unclear. It is possible that the patients actually had AF, but that this was not noted anywhere in the doctors’ or nurses’ reports. Another explanation would be that the measurements were disturbed by the patients’ movements or that the watch was not worn correctly.

There are some wearables available with higher accuracy compared to the Scanwatch (17). The Apple Watch usually performs very well. They achieve a sensitivity of over 95% and a specificity of over 80% (18) and Perez et al. showed a PPV 78% (19). But similarly good values can also be seen in the KardiaBand from Alivecor with a sensitivity of 93% and a specificity of 84% (20) and in the devices from Honor and Huawei (21). However, all these studies differed in their design. But there are also studies that have examined smartwatches and their ability to recognise AF in patients after an operation. For example, Inui et al. analysed devices from Fitbit and Apple and showed that the Apple Watch was more effective (22). Compared to our results, Müller et al. showed approximately 22% higher sensitivity and 3% lower specificity of the Apple Watch in patients with POAF (23). Although the studies are very similar, there are some differences, such as the detection method (ECG vs. PPG) or the observation period. However, there is not prospective head-to-head comparison with the Scanwatch.

With a sensitivity of 69%, episodes of AF can obviously be missed by the Scanwatch. This in turn can lead to serious complications. Potential strategies to improve the Scanwatch's detection accuracy include the following considerations. The two easiest ways would be to increase the frequency of measurements and reduce the number of suspicious measurements needed to receive a notification by the app. Both could have a positive effect on sensitivity. To further increase specificity, the ECG function of the Scanwatch could be utilised after it has detected AF using the PPG sensor. The disadvantages here, would be the increased battery consumption due to the increased number of measurements and possibly more false positive results. It could therefore be a compromise if these two variables could be customised. In addition advanced algorithms and modelling, such as deep learning could help to improve detection accuracy and should be the aim of future studies. It is important to note that the Scanwatch PPG is not designed for definitive AF diagnosis but rather as a screening tool. Any positive screening result from the smartwatch should always be followed by further verification. This can be achieved either through a single-lead ECG provided by the watch itself or by seeking confirmation from a medical professional using a 12-lead ECG or extended ECG monitoring. This step is essential to accurately confirm or rule out the diagnosis of AF. This approach is also strongly supported by the current European Society of Cardiology (ESC) guidelines for the diagnosis and management of AF, which emphasize the need for thorough validation following initial AF detection (11).

The Scanwatch's appeal extends beyond its AF screening capabilities, which are notable for their high specificity and moderate sensitivity. It also has a longer battery life compared to many of its competitors and features a user-friendly design that operates solely through a single crown. This simplicity is particularly advantageous for older individuals who may find technology-intensive wearables challenging to use, yet are at a higher risk for AF. The straightforward operation of the Scanwatch ensures ease of use while providing valuable health monitoring for this vulnerable demographic. Some studies indicate that given a choice, patients lean towards the Scanwatch over other smartwatches (14) or wearable devices (15).

It is plausible that, in the near future, smartwatches and other wearable devices will become instrumental in screening for AF, both after cardiac surgery and in the everyday lives of individuals at increased risk for the condition. While the monitored hospital setting may not fully represent everyday conditions, it is conceivable that smartwatches and wearables could enable earlier detection of AF. These devices might help in preventing complications associated with POAF, such as strokes and premature death (7). This becomes increasingly important because POAF can recur after its initial resolution or may develop after discharge (9, 10). The viability and cost benefits of extended POAF screening should be the subject of upcoming research. Nevertheless, existing studies indicate that systematic AF screening for the elderly is effective (24), and smartwatch-based methods are cost-efficient (25). Given these advantages, health insurance providers and hospitals might contemplate providing high-risk patients with smartwatches for AF screening, aiming to curtail both complications and expenses. However, the specific role of AF burden in this context remains insufficiently defined. There is limited evidence in this regard, but we assume that a high AF burden is associated with an increased number of complications and comorbidities. In contrast, only short episodes of AF could be of marginal or no clinical relevance.

It is important to emphasize that wearables should not and cannot replace telemetry as the gold standard. Telemetry not only excels in detecting AF but is also superior in identifying other potentially fatal cardiac arrhythmias. To illustrate, based on data from our study sample depicted in Figure 2, the Scanwatch underestimated heart rates after the AF onset and the values are measured incorrectly. But, it did detect AF, registering irregular and elevated pulses at multiple instances, as depicted in a comparative view with telemetry data in Figure 3. One possible explanation would be that the PPG was not able to detect all beats in each of the measurements and therefore estimated the heart rate incorrectly. This question cannot be conclusively answered without the exact PPG data and the algorithm used to evaluate the data. Still, devices like the Scanwatch gain relevance when patients regain mobility. A notable advantage of the Scanwatch over telemetry is its enhanced comfort for the patients and users.

The use of the Scanwatch may also support the application of pharmacological cardioversions through the “pill in the pocket” method. Here, upon detecting and confirming an AF episode using wearable or standard ECG, antiarrhythmic drugs like flecainide or propafenone are used to restore sinus rhythm. This treatment strategy is already endorsed in the ESC guidelines, though it excludes patients who recently experienced acute coronary syndrome or those with severe heart failure (11).

**Figure 4:**
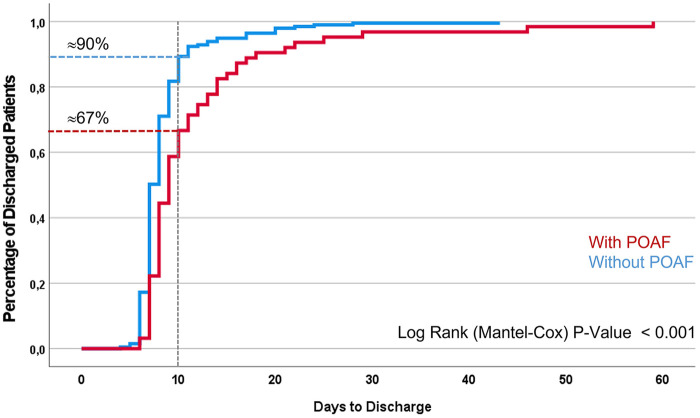
Time to discharge*.*

Our study found that AF was more common in the early days following surgery, particularly among older patients with higher CHA2DS2-VA-Scores and those undergoing multiple surgeries. While specific diagnoses did not show statistical significance—likely due to the characteristics of our study population and the focus on POAF that developed shortly after cardiac surgery—our results confirm that older age and multimorbidity are significant risk factors for POAF. Therefore, patients with these characteristics could benefit from more intensive and extended AF screening. Additionally, our findings revealed that patients with POAF experienced a notably longer time to discharge, as illustrated in Figure 4. Although these results are consistent with existing knowledge, they serve to reinforce and validate previous findings (7).

### 4.1 Limitations

Our study has several limitations. One key limitation is the relatively small sample size of 32 patients who experienced AF in the early days following surgery. Additionally, we did not personally review all the telemetry data, which raises concerns about the accuracy of the rhythm evaluations performed by medical staff and the potential for missing brief AF episodes. This potential inaccuracy of the gold standard may therefore also affect the sensitivity and specificity of the Scanwatch. Particularly, episodes of normofrequent AF might not have been captured by the telemetry. Lastly, the functionality of the Scanwatch itself presents a limitation. The device measures pulse using a PPG sensor at fixed 10-minute intervals, which cannot be adjusted. Consequently, short AF episodes may have gone undetected by the device. In addition, we unfortunately did not have access to more precise data or the algorithm of the Scanwatch and were unable to implement or test any improvements.

## 5 Conclusions

In this clinical validation of a smartwatch, we found that using it to screen for AF in patients after cardiac surgery is both reliable and feasible. Our study demonstrated that the Withings Scanwatch is highly accurate in detecting AF using its PPG sensor under clinical conditions. However, while smartwatches and wearables are not a substitute for traditional screening methods such as telemetry and 12-lead ECG, they can complement these methods and enhance their effectiveness. Further research is needed to assess how continuous monitoring with wearables affects complication rates, patient outcomes, and associated healthcare costs.

## Data Availability

The raw data supporting the conclusions of this article will be made available by the authors, without undue reservation.
