# Correction: Feasibility and reliability of a smartwatch to detect atrial fibrillation after cardiac surgery: a prospective study

**DOI:** 10.3389/fdgth.2026.1818164

**Published:** 2026-03-16

**Authors:** Konrad Schreier, Michael Borger, Alireza Sepehri Shamloo, Lukas Hofmann, Thomas Schröter, Sandra Eifert, Angeliki Darma, Christian Etz, Sergey Leontyev, Martin Misfeld, Andreas Bollmann, Arash Arya

**Affiliations:** 1 Department of Cardiac Electrophysiology, Heart Center Leipzig, Leipzig, Germany; 2 Department of Cardiac Surgery, Heart Center Leipzig, Leipzig, Germany; 3 Department of Cardiothoracic Surgery, Royal Prince Alfred Hospital, Australia Institute of Academic Surgery, Sydney, NSW, Australia; 4 The Baird Institute of Applied Heart and Lung Surgical Research, Australia Sydney Medical School, University of Sydney, Sydney, NSW, Australia; 5 University Clinic and Polyclinic for Cardiology, University Hospital Halle (Saale), Martin-Luther University Halle-Wittenberg, Halle, Germany

**Keywords:** atrial fibrillation, cardiac surgery, monitoring, screening, smartwatch

Author Arash Arya was erroneously assigned as corresponding author. The correct corresponding author is Konrad Schreier.

The original version of this article has been updated.

